# Minimal invasive nonfusion technique for the treatment of noncontiguous lumbar burst fractures in young age patient: A case report

**DOI:** 10.1097/MD.0000000000010009

**Published:** 2018-03-09

**Authors:** Jae-Kwang Kim, Bong Ju Moon, Sang-Deok Kim, Jung-Kil Lee

**Affiliations:** Department of Neurosurgery, Chonnam National University Hospital and Medical School Gwangju, Republic of Korea.

**Keywords:** noncontiguous burst fracture, short-segment pedicle screw fixation, spinal canal decompression, temporary

## Abstract

**Rationale:**

In the treatment of noncontiguous lumbar burst fractures, there still remains controversy over proper surgical procedures.

**Patient concerns:**

A 19-year-old female patient visited our hospital after fall down from 3 m high.

**Diagnoses:**

Initial neurologic examination revealed an incomplete spinal cord injury characterized by hypoesthesia and motor grade of 2 below the L2 segment. Lumbar computed tomography and magnetic resonance imaging demonstrated L2 and L5 burst fractures severely obliterating the spinal canal.

**Interventions:**

She underwent emergent PSSPSF at L1-2-3 and L4-5-S1 following bilateral L1 and L4 laminotomy with reduction of bony fragments by tapping method.

**Outcomes:**

She was gradually recovered and able to walk with assistance two weeks after surgery. Removal of implants was performed at 12 months after surgery. Follow-up radiography showed well-preserved segmental motion and adequate decompressed spinal canal with fused fractured bony fragment. She returned to her normal daily activities without any neurologic deficits and pain.

**Lessons:**

Noncontiguous burst fracture of the lumbar spine is an unusual injury. For the adequate management in patient with neurologic deficit, reduction of the fractured body and stabilization of vertebral column is necessary. It is also important to preserve the segmental motion in young age patients. From that point of view, temporary PSSPSF with spinal canal decompression is considered as minimal invasive surgery with significant low morbidity, providing stability with motion saving and good clinical outcome.

**Key points**

1. Noncontiguous multiple lumbar burst fractures is a rare clinical entity that causes severe neurologic deficit.
2. Especially in the treatment of young age patient, motion saving is very important factor.
3. Minimal invasive nonfusion surgery can be effective treatment option for the young age noncontiguous lumbar burst fractures.

## 1 Introduction

Noncontiguous multiple lumbar burst fractures is relatively rare accounting for approximately 5% of all spinal burst fractures.^[1]^ In many studies, noncontiguous burst fractures are high energy fractures, and surgical treatment has been reported to be superior to conservative treatment if neurologic abnormalities are present. There still remains considerable controversy between various surgical procedures including short-segment or long-segment screw fixation, anterior or posterior decompression through corpectomy and combined approach for the treatment of burst fractures.^[2–4]^ Surgeon's preference is important which approach is determined according to the fracture type or neurologic deficit because there is lack of evidences for one approach over the others.^[5]^ In terms of minimal invasive surgery, PSSPSF involving one level above and below the fractured vertebra has recently become popular in the treatment of thoracolumbar and lumbar burst fractures due to its low morbidity compared to standard midline posterior approach or anterior approach. In this report, we present a 19-year-old young patient of a simultaneous L2 and L5 burst fractures who treated with temporary PSSPSF following spinal canal decompression.

### 1.1 Case description

A 19-year-old female patient presented with history injured by falling down from a second floor of approximately 3 meters high. At initial evaluation, she suffered from severe low back pain with numbness and weakness on both legs. Neurologic status was assessed using the American Spinal Injury Association (ASIA) impairment scale. An incomplete spinal cord injury characterized by hypoesthesia and motor grade of 2 below the L2 segment was demonstrated. (ASIA scale C). She also suffered from urinary retention required bladder catheterization and perianal numbness indicating injury of conus medullaris. But anal sphincter tone and continence was maintained.

### 1.2 Radiologic findings

**Figure 1:**
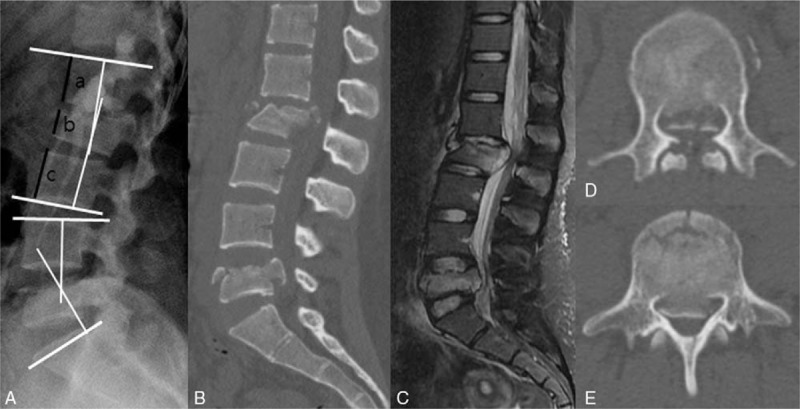
Preoperative radiologic analysis. (A) The Cobb angle of L2 and L5 was formed between white lines were 3.8° and −32.8°, respectively. The vertebral body compression calculated by the formula being [1−(2b/a + c)] × 100. Calculation results were 40% at L2 and 50% at L5. (B) The preoperative sagittal CT scan showed severe obliteration of the spinal canal. (C) Preoperative MRI scan demonstrated traumatic disc injury at L1-2 and acute burst fractures at L2 and L5. Before surgery, canal compromise of L2 (C) and L5 (D) was 70% and 50%, respectively. CT = computed tomography, MRI = magnetic resonance imaging.

Supine anteroposterior and lateral radiographs, CT and MRI scan were performed before surgery. A initial CT and MRI scans revealed noncontiguous both L2 burst fracture with 40% vertebral body compression, 70% canal compromise and suspicious tear of ligamentum flavum (AO classification, B1) and L5 burst fractures with 50% vertebral compression, 50% canal compromise (AO classification A3) (Fig. 1B–D). Regional kyphosis measured as Cobb method was 3.8° and −32.8° at L2 and L5, respectively (Fig. 1A). The TLICS and LSC were all 7.

### 1.3 Surgical techniques

**Figure 2:**
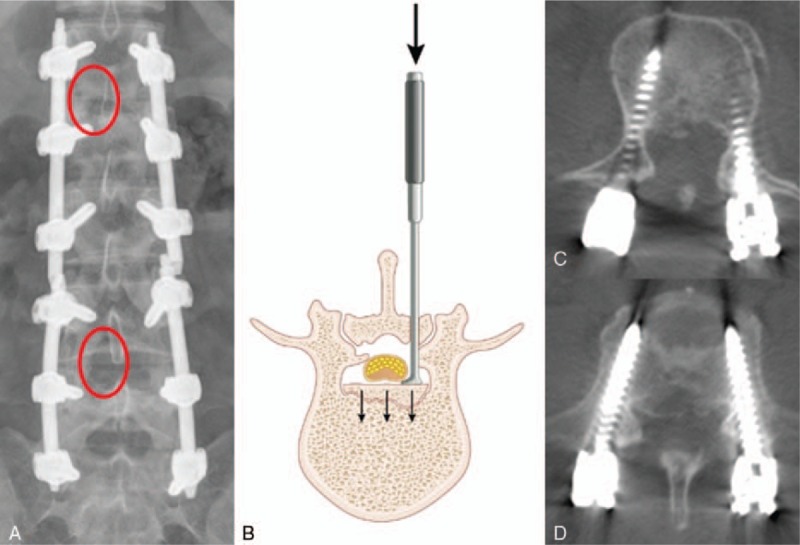
(A) In the postoperative radiography, the two red circles indicate the area of laminotomy. (B) The illustration described tapping and reduction technique of the bony fragments severely compromising the spinal canal. Postoperative 12 months follow-up CT scans showed well-fused fractured bony fragments and canal compromise was reduced from 70% to 28% at L2 (C) and 50% to 30% at L5 (D). CT = computed tomography.

The midline posterior approach was performed for spinal canal decompression prior to screw fixation under general anesthesia. Minimal skin incision and muscle dissection was carried out for exposure of lamina on L1 and L4. After laminotomy on both L1 and L4, careful retraction of dural sac and tapping of the bony fragments which compromise the spinal canal. (Fig. 2A and B). Under fluoroscopy, pedicle screws were inserted with Sextant (Medtronic Sofamor Danek, Tennessee) system into the vertebral body one level above and below the fractured vertebra as well as fractured vertebral body (L1-2-3 and L4-5-S1 screw fixation). All screws were connected with rods which were slightly bent in to a lordotic curve. Distraction or cross-link was not applied.

### 1.4 Radiologic outcomes

**Figure 3:**
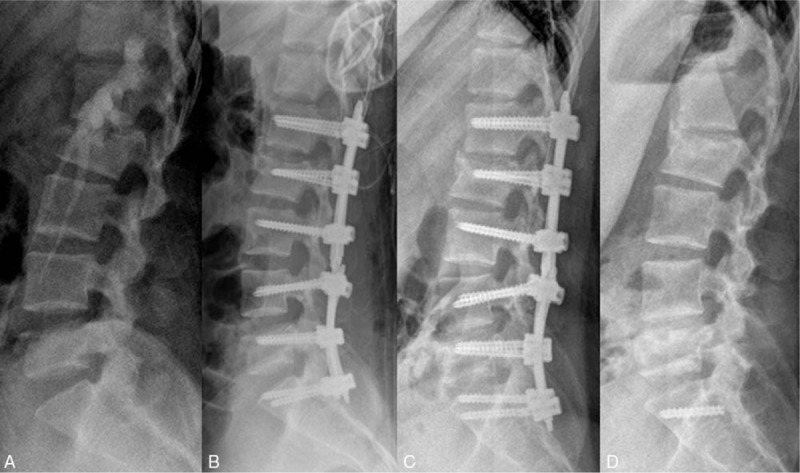
Change of Cobb angle in serial lateral radiographs. (A) Before surgery, the Cobb angle of L2 was 3.8° and that of L5 was −32.8° (B) After immediate surgery, the angle was reduced to −7.5° and −36.1° (C) At 12 months after surgery, the angle changed to 6.1° and −15.6° (D) At 24 months after surgery, the angle increased to 13.3° and −15.5°.

A radiologic assessment was performed using standing anteroposterior and lateral radiographs and CT scans immediate postoperatively, 12 months postoperatively (at the time of implants removal) and 24 months postoperatively (at 12 months after implants removal). At 12 months after surgery, there occurred a screw breakage at left-side S1 but bony fusion of fracture site was successfully achieved at both L2 and L5 where tapping and reduction of the bony fragments. Also, canal compromise was reduced from 70% to 28% at L2 and 50% to 30% at L5. (Fig. 2C) The regional kyphosis was corrected from 3.8° before surgery to −7.5° after surgery at L2 and from −32.8° to −36.1° at L5. It gradually increased at both L2 and L5 and the correction loss was 20.8 and 20.6, respectively, at 24 months after surgery (Fig. 3).

### 1.5 Clinical outcomes

LBOS was used to assess the functional outcome. She was gradually recovered and able to walk with assistance 2 weeks after surgery. (LBOS 14, poor) Thoracolumbosacral brace was applied for 2 months. At 12 months after surgery, we decided to remove the implants because the pain completely resolved with motor grade of more than 4 and bony fusion was achieved. (LBOS 35, fair) At 24 months after surgery, although regional kyphosis slightly increased, she returned to her normal daily activities without any neurologic deficits and pain (LBOS 56, good).

## 2 Discussion

Surgical treatment has significant advantages of providing immediate stability and correcting kyphosis in the treatment of noncontiguous lumbar burst fractures compared to nonsurgical treatment.^[6]^ The surgical considerations of lumbar burst fractures include fracture type, fracture location, posterior ligament injury, and preoperative neurologic deficit. But controversy remains concerning the proper management of lumbar burst fractures. Then, is fusion necessary for thoracolumbar and lumbar burst fractures? Fusion could provide spinal column stability, correction of regional kyphosis, low incidence of implant failure.^[7]^ But it is significantly associated with intraoperative and postoperative morbidity such as longer operation time, iatrogenic muscle trauma, blood loss, infection, postoperative pain or adjacent segment degeneration.^[6,8,9]^

A recent meta-analysis reported that no significant difference between the fusion and non-fusion groups regarding radiologic and functional outcomes.^[10]^ Wang et al^[9]^ also reported that short segment screw fixation without fusion was satisfactory in the view of clinical outcomes and implant failure.

In this case of AO type A3 (L2) and B1 (L5), LSC 7 noncontiguous lumbar burst fracture we used temporary PSSPSF with bilateral laminotomy and tapping of bony fragments at each level. PSSPSF become popular for thoracolumbar and lumbar burst fractures. We already reported 42 patients of thoracolumbar burst fractures treated with PSSPSF and laminotomy.^[11]^ There were no implant failure and spinal column instability requiring reoperation. Additional screws into the fractured vertebra could indirectly support anterior column and reduce implant failure compared to traditional short segment screw fixation, one level above and below the fractured vertebra. The patient showed a good correction of kyphosis immediately after surgery but kyphosis had slightly progressed at both L2 and L5 after implant removal. We thought that it was inevitable due to traumatic injury of L1-2 disc. Adjacent disc injury at onset followed by degeneration might accelerate loss of disc height and thus kyphosis progressed after implant removal. So it might be a limitation of temporary PSSPSF. But the relationship between kyphosis and outcome is not clear.^[12,13]^ Although the kyphosis had progressed during follow-up in this case, it was not related to persistent back pain or neurologic deficits.

Beside of PSSPSF with laminotomy, tapping of the bony fragments was added due to severe compromise of the spinal canal with motor weakness. On follow-up CT scan, we confirmed successful bony fusion at 12 months after surgery with marked improvement of spinal canal compromise. Removal of implants was performed because bony fusion of fractured vertebra indicates complete healing and restoring vertebral column stability. Moreover, preservation of segmental motion is more important to especially young patients than restriction of the range of movement with rigid fixation which might make young patient discomfort.

## 3 Conclusions

Young age noncontiguous lumbar burst fractures is unique manifestation. Temporary PSSPSF with tapping technique could be an effective independent treatment modality in the treatment of noncontiguous lumbar burst fractures, especially in young patients who need to preserve the motion and return to normal daily activity.
